# Early Aβ42 Exposure Causes Learning Impairment in Later Life

**DOI:** 10.14336/AD.2021.1015

**Published:** 2022-06-01

**Authors:** Kuan-Chung Cheng, Chun Hei Antonio Cheung, Hsueh-Cheng Chiang

**Affiliations:** ^1^Department of Pharmacology, National Cheng-Kung University, Tainan, Taiwan.; ^2^Institute of Basic Medical Sciences, College of Medicine, National Cheng-Kung University, Tainan, Taiwan

**Keywords:** Aβ, learning and memory, XBP1, oxidative stress, aging

## Abstract

Amyloid cascade hypothesis proposes that amyloid β (Aβ) accumulation is the initiator and major contributor to the development of Alzheimer’s disease (AD). However, this hypothesis has recently been challenged by clinical studies showing that reduction of Aβ accumulation in the brain does not accompany with cognitive improvement, suggesting that therapeutically targeting Aβ in the brain may not be sufficient for restoring cognitive function. Since the molecular mechanism underlying the progressive development of cognitive impairment after Aβ clearance is largely unknown, the reason of why there is no behavioral improvement after Aβ clearance remains elusive. In the current study, we demonstrated that transient Aβ expression caused learning deficit in later life, despite the accumulated Aβ was soon being removed after the expression. Early Aβ exposure decreased the cellular expression of XBP1 and both the antioxidants, catalase, and dPrx5, which made cells more vulnerable to oxidative stress in later life. Early induction of XBP1, catalase, and dPrx5 prevented the overproduction of ROS, improved the learning performance, and preserved the viability of cells in the later life with the early Aβ induction. Treating the early Aβ exposed flies with antioxidants such as vitamin E, melatonin and lipoic acid, after the removal of Aβ also preserved the learning ability in later life. Taken together, we demonstrated that early and transient Aβ exposure can have a profound impact on animal behavior in later life and also revealed the cellular and molecular mechanism underlying the development of learning impairment by the early and transient Aβ exposure.

It is long believed that amyloid β (Aβ) peptide accumulation induces memory loss. The Aβ hypothesis proposes that Aβ accumulation in the brain initiates a series of signaling cascade, which triggers neuronal dysfunction and cell death. However, this hypothesis has recently been challenged by findings showing that reduced accumulation of Aβ in the brain does not produce significant improvement on cognitive function in patients with AD [1, 2], raising a question on the role of Aβ accumulation in the development of this disease, and also suggesting that an unidentified risking factor that can affects memory performance, during or after Aβ clearance, may be present. While the cause of memory dysfunction after the clearance of Aβ remains elusive, the pathologies between early Aβ accumulation and post-Aβ clearance are also largely unknown.

Traumatic brain injury (TBI) has been proposed as one of the risking factors for AD [3, 4]. Early TBI experience increases the chance of developing dementia in later life [5]. In addition, it has been shown that TBI increases acute Aβ accumulation in the region of injury, suggesting a possible cause-effect relationship between the early Aβ accumulation and the late dementia occurrence [6, 7]. Interestingly, the increased Aβ accumulation after brain injury is not always elevated, in the PDAPP mice, familial AD mutation V→F at amyloid precursor protein (APP) position 717, there is increased Aβ42 2 hours after brain trauma but the level returns to the basal level 6 hours after injury [8]. Studies from TBI patients has shown that the accumulation of Aβ42 reached the peak within one week after injury and then gradually decreased to the levels that are similar to the basal levels of the control group [9]. Furthermore, different results have been reported in a study examining the association between amyloid burden and hippocampal atrophy after TBI. Increased hippocampal atrophy, but with reduced amyloid burden, were observed in the PDAPP mice after injury [10]. Declined CSF Aβ concentration has also been reported to be correlated with the worsened clinical status in TBI patients [11]. Therefore, even though the molecular mechanism underlying these differential results remains unclear, these findings suggest that transient and short exposure of Aβ accumulation as an early event may trigger pathological changes in later life.

Accumulating evidence has suggested a role of ER stress in the development of AD [12]. Activation of protein kinase RNA-like ER kinase (PERK) and X-box binding protein 1 (XBP1) has been found in AD patients, as compared to the nondemented people [13]. Increased XBP1 splicing reverses the spatial memory in the AD mice [14], prevents the death of neurons, and extends the life-span of the Aβ42 flies [15, 16]. Interestingly, studies showed that XBP1 is transiently increased in the early stage of learning and memory impairment in the APP/ presenilin 1 (PS1) and 5xFAD mice [17], Aβ transgenic flies [16], and human AD patients [17]. Despite activation of XBP1 has been considered as a protection for cells and early induction of XBP1 could be a cell protective response against Aβ toxicity, chronic stresses promote the reduction of XBP1 activity [12].

Oxidative stress is known to play a role in the development of numerus diseases including AD [18, 19]. Increased ROS production or accumulation has been shown to trigger various molecular and cellular abnormalities such as DNA oxidation, lipid oxidation, and mitochondria dysfunction [19, 20]. Early accumulation of oxidative stress is observed in the early stage of AD and mild cognitive impairment (MCI) [21, 22]. Early accumulation of ROS during oligomer formation before plaque and tangle formation could also be found in the triple transgenic mice harboring PS1(M146V), APP(Swe), and tau(P301L) transgenes [23]. Behavioral recovery experiments showed that reducing oxidative stress in the brain, through both genetic and pharmacological approaches, improves the cognitive performance in the AD animal model [24, 25]. However, despite growing amount of evidence has suggested a relationship between the Aβ accumulation and the oxidative stress induction, the underlying mechanism remains elusive.

It is believed that the molecular mechanism and the process of memory formation are highly conserved among different species [26]. The similarity of pathological phenotypes in between the Aβ42 flies and mammals suggests that the Aβ toxicity may also preserved in the Aβ42 flies [16, 27, 28]. In the current study, we showed that early and transient accumulation of Aβ affected later learning and memory performance in flies. Transient Aβ accumulation in the early stage decreased the expression of ER response and antioxidant genes and increased the levels of ROS in later life. Importantly, antioxidant treatments lowered the ROS levels and prevented the development of the learning and memory impairment in flies. Our data, for the first time, not only reveals the pathological role of the early and transient Aβ accumulation in the development of learning and memory impairment but also partially validates the Aβ hypothesis. Besides developing novel Aβ detector or finding biomarkers to indicate the early status of disease, this study offers a new alternative way to investigate the medical intervention for the future disease treatment, a combination of current Aβ clearance strategy and antioxidant application.

## MATERIALS AND METHODS

### Fly Stocks

All flies were raised on standard food at 18°C after crossed, then transferred to 30°C until adult. Besides, all flies were stored under normal circadian cycle (12:12 light dark cycle).

Elav-Gal4 were used as pan-neuronal driver for all experiments. Following fly stocks were come from the Bloomington Drosophila Stock Center (BDSC): Tublin-Gal80^TS^ (BDSC 7017), UAS-dFOXO (BDSC 42221), UAS-Rac1 DN (BDSC 6292), UAS-EGFR DN (BDSC 5364), UAS-JNK DN (BDSC 9311), and UAS-Catalase (BDSC 24621). Elav-gal4, UAS-Aβ42 were gifts from Dr. Yi Zhong at Tsinghua University, China. UAS-XBP1d08698 was a gift from Dr. Pedro Fernandez-Funez at the University of Texas Medical Branch, USA. UAS-GADD34 was a gift from Dr. Stefan Marciniak, at the University of Cambridge, UK. UAS-dPrx5 was a gift from Dr. William C. Orr, at the Southern Methodist University, USA.

### Pavlovian Olfactory Aversive Conditioning

All experiments were done in a 27°C room with 70% relative humidity as described previously [29, 30]. During training process, about 100 flies were put in training tube containing electric path where two aversive odors, 3-octanol (OCT, Sigma-Aldrich) and 4-methylcyclohexanol (MCH, Sigma-Aldrich), were given to flies. The odor concentrations were adjusted to avoid bias by daily condition. The flies first exposed to the odor paring with electric shock (12 pulses of 90 V per 1 minute) for 1 minute. The odor paring with electric shock was called CS+ odor. Then, through 45 seconds interval of fresh air, flies exposed to the second odor without electric shock. The odor without electric shock was called CS- odor. One training process included one CS+ and one CS- odor exposure.

During test process, for immediate memory measurement, we immediately send the trained flies into a T-maze where flies could choose between CS+ and CS- for 2 minutes. The measurement of immediate memory also referred to as learning. For short term memory (STM) measurement, flies were placed in the same food containing vial they had been kept in before training process until the test. Two hours after training, flies were examined for STM. The distribution of flies in the two T-maze arms is referred to as performance index (PI) [29]. A reciprocal group of flies was trained and tested by using OCT as the CS+ and MCH as the CS+, respectively. The data were finally averaged for one data (n = 1) and multiplied by 100. The wonderful learning was performed as PI of 100, indicating all flies avoided the CS+ odor. However, a PI of 0 indicated the equal distribution of two odors, referring to no memory retention. Control groups were age-matched to the experimental groups in each test.

### Western Blot Analysis

Five fly heads were homogenized in SDS sample buffer (LC2676, Invitrogen). Lysates were centrifuged. The resulting supernatant was collected, and proteins were resolved on 15% Tris-tricine gels. The protein samples were transferred to nitrocellulose membranes with 0.22 µm pore size (Pall Corporation). The membranes were blocked with 5% nonfat dry milk and incubated with anti-β-Amyloid (1:2000, #8243 CST), anti-GABARAP (1:2000, #13733 CST), anti-Ubiquitin (1:2000, #3936 CST), anti-Ref2P (1:2000, ab178440 Abcam) and anti-GAPDH (1:50000, GTX100118 GeneTex) primary antibodies in TBST at 4°C overnight. The rabbit horseradish peroxidase-conjugated secondary antibody was used, and the signal was detected by chemiluminescence. The signal intensity was quantified by Image J software.

### Survival Assay

Flies were collected under light CO_2_ anesthesia and divided into vials containing regular food. Flies were passed onto fresh food vials every 3-4 days and the number of dead flies were recorded. The experiments were placed in 30°C, with 70% humidity, on a 12-h light/dark cycle and performed at least twice. All results were analyzed from experiments carried out using female flies.

### Quantitative RT-PCR

Trizol was used to isolate total RNA from 20 fly heads. Then, RevertAid RT Reverse Transcription Kit (K1691, Thermo Scientific) was used to eliminate DNA traces. Finally, One Step RT-PCR (Applied biosystems) was used to prepare cDNAs. For detection of *Drosophila XBP1s*, the primers dXBP1s550F 5’-ACCAACCTTGGATCTGCCG-3’ and dXBP1s697R 5’-CGCCAAGCATGTCTTGTAGA-3’ were used to amplify a 140 bp fragment as previously described [15]. For quantification of the expression of *XBP1* in *Drosophila*, the primers XBP1t-F 5’-TGGGAGG AGAAAGTGCAAAG-3’ and XBP1t-R 5’-TCCGTTC TGTCTGTCAGCTC-3’ were used to amplify 125 bp fragment in the 5’ exon [15]. For detection of *dPrx5*, the primers dPrx5-F 5’-AGAGAGGAGAAATGCGTGTG-3’ and dPrx5-R 5’-CACTGGTTTTGGACAGGGAT-3’ were used to amplify a 105 bp fragment. For quantification of the expression of *catalase* in *Drosophila*, the primers Cat-F 5’-CTCTGATTCCTGTGGGCAAA-3’ and Cat-R 5’-AGTAGGAGAACAGACGACCA-3’ were used to amplify a 150 bp fragment. As internal control, the primers for *dRpl32*: dRpl32-F 5’-AATCCTCGTTGG CACTCACC-3’ and dRpl32-R 5’-TGTTGTGTCCTT CCAGCTTCA-3’ were used to amplify a fragment of 135 bp. For qPCR with the SYBR Select Master Mix (#4472913, Applied biosystems) and StepOnePlus™ Real-Time PCR System (Thermo Fisher), we used 10 ng of purified RNA. Cycling conditions were set holding state at 50°C for 2 min, 95°C for 2 min and 40 cycles of 95°C for 15 s, 56°C for 1 min and 72°C for 1 min. The levels of RNA were calculated using the StepOne Software v2.3 (Thermo Fisher), which relied on the comparative Ct method of quantification. All mRNA levels were normalized with *dRpl32*.

### Propidium iodide staining

Fly brains were quickly dissected in PBS and fixed in 4% paraformaldehyde in PBS overnight. Then, after vacuumed 3 times for 20 minutes each, the brains were permeabilized with 2% TritonX-100 in PBS overnight. Next, fly brains were incubated with 1:200 propidium iodide in PBS at 4 °C overnight., The brains were washed with PBS 3 times for 20 minutes and subsequently mounted between two glass coverslips in focusclear™, and imaged on a FluoView FV1000 confocal microscope.

### Antioxidant Feeding

Lipoic acid (T5625, Sigma-Aldrich) and melatonin (M5250, Sigma-Aldrich) were diluted in ethanol and mixed with the regular food to make the final concentration of 2 mM and 100 μg/ml respectively. Vitamin E (#258024, Sigma-Aldrich) was made in the final concentration of 20 μg/ml. All flies were transferred to fresh tubes every 3-4 days to avoid food desiccation and compound degradation.

### Starving stress resistance

To trigger starving stress, flies were placed in the vials containing with filter paper moistened with water. The papers were replaced every 12 hours. The survival of flies was recorded every several hours and kept under stress condition until whole flies died. Each experiment was performed at least twice.

### Oxidative stress resistance

To trigger oxidative stress, flies were kept on filter paper soaked with 40 mM Methyl Viologen (Paraquet, #856177, Sigma-Aldrich) in 4% sucrose solution in the vials. The papers were replaced every 12 hours. The survival of flies was recorded every several hours and kept under stress condition until whole flies died. Each experiment was performed at least twice.

### H2DCF staining

2′,7’-dichlorofluorescein (H2DCF, D6883, Sigma-Aldrich) was used to detect ROS following previously described protocols [31]. Briefly, fly heads were dissected in PBS, then placed in 10 μM H2DCF for 15 minutes at room temperature. Before mounting, samples were washed three times for 5 minutes each in PBS at room temperature on a shaker. Brains were mounted between two glass coverslips in focusclear™ and imaged on a FluoView FV1000 confocal microscope.

### Proteasome Activity Assay

Proteasome activity was measured using a Proteasome Activity Fluorometric Assay Kit (K245, Biovision). According to the manufacturer instruction, five fly heads were homogenized in 0.5 % NP-40 in dH_2_O and suspended in assay buffer. Then, samples were incubated with an AMC-tagged peptide substrate for 30 minutes at 37° C to produce fluorophore. Finally, the fluorescence was measured by using an ELISA plate reader (SpectraMax iD3 Multi-Mode Microplate Readers, Molecular Devices) at Ex/Em = 350/440 nm in kinetics mode at 37°C for 30-60 minutes.

### Image quantification

We used FV1000 analysis software and Image J software to analyze all the images. Statistical analysis was performed on samples from three or more brains.

For cell loss measurement, the quantified vacuolated areas within cell body region were divided by total area of cell body region around calyx. The final data was presented as the percentage of cell loss. To prevent the bias, we used double blind to analysis our data.

For H2DCF staining, we stacked all frames and used the mean of the summed H2DCF intensities averaged from whole brain for statistical analysis. Three brain intensities within one experiment were finally averaged for one data (n = 1) and we analyzed at least three experiments.

### Statistics

All data were analyzed using Graphpad Prism 6.0 software. Comparisons of multiple groups used one-way ANOVA followed by Tukey’s multiple comparison test. Comparisons between two groups used two-tailed t-test. Statistical significance was shown with *P* value <0.05. Statistical results were presented as means ± SEM.

## RESULTS

### Reduction of Aβ accumulation temporary reverses Aβ-induced behavioral damages

**Figure 1.**
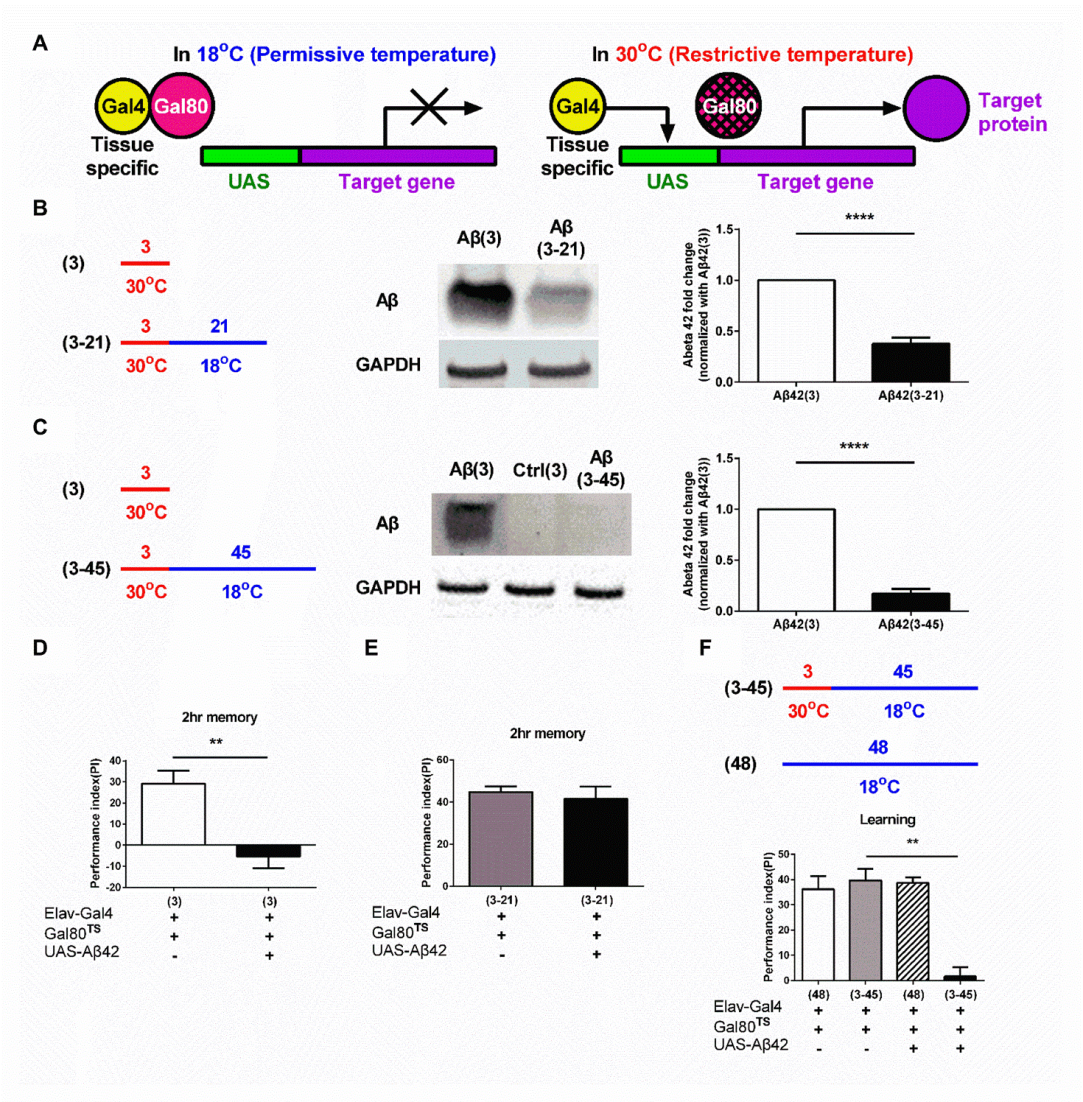
The Aβ-induced learning and memory deficits are temporally improved by Aβ clearance. (A) Under the permissive temperature, Gal80^TS^ protein binds to Gal4 protein to repress the transcriptional activity of GAL4; thus, preventing the expression of the target protein. Under the restrictive temperature, the conformation of Gal80^TS^ protein is changed, releasing, and allowing the GAL4 protein to drive the expression of the target protein. (B) The Aβ level in the brain of 3_30_°_C_-21_18_°_C_ flies was reduced, as compared to 3_30_°_C_ flies. Results of the respective quantitative analysis is shown on the right, N = 8 respectively. ****p < 0.0001. (C) The Aβ level in the brain of 3_30_°_C_ -45_18_°_C_ flies was almost undetectable. The quantitative data is shown on the right, N = 4 respectively. ****p < 0.0001. (D) Overexpression of Aβ for 3 days induced memory deficit, N = 6 respectively. ** *p* < 0.01. (E) The memory deficit in 3_30_°_C_ flies was reversed in 3_30_°_C_ -21_18_°_C_ flies, N = 6 respectively. (F) Learning impairment was reoccurred in 3_30_°_C_ -45_18_°_C_ Aβ flies (Ctrl (3-45) vs. Aβ42 (3-45): *p* = 0.0041, N = 6 respectively). ** *p* < 0.01. All Ctrl represents Elav-Gal4+Gal80^TS^.

We employed conditional expression system, Gal80^ts^ [32], to temporary regulate the Aβ42 expression. Gal80^ts^ is the temperature-sensitive version of Gal80. Unless mentioned otherwise, all the experiments were done with using *Elav-Gal4* promoter to induce Aβ expression in neurons (Fig. 1A). We demonstrated in a previous study that memory deficit can be observed 3 days after Aβ expression in the adult Aβ42 flies [16]. To prove that the accumulated Aβ (3 days post-induction) can be removed in the brain of fly; we transferred the flies from the restrictive temperature, 30°C, to the permissive temperature, 18°C, to terminate the Aβ expression process and were subsequently housed under the permissive temperature for 21 days (*i.e.*, 3_30_°_C_-21_18_°_C_ Aβ42 flies), we examined the Aβ level in the brain of 3_30_°_C_-21_18_°_C_ Aβ42 flies. As shown in Fig. 1B, the amount Aβ accumulated in the brain of 3_30_°_C_-21_18_°_C_ Aβ42 flies was decreased, as compared to 3_30_°_C_ Aβ42 flies (*i.e.*, only with 3 days induction) (P < 0.0001, unpaired Two-tailed T-test). Flies with 45 days post-housing under the permissive temperature after 3 days of Aβ induction (*i.e.*, 3_30_°_C_-45_18_°_C_ Aβ42 flies) showed very few Aβ left in the brain (Fig. 1C; P < 0.0001, unpaired Two-tailed T-test). Notably, Aβ flies only housed under the permissive temperature for 48 days had no Aβ expression, confirming that the *UAS-*leakage under the permissive temperature is negligible (Supplementary Fig. 1). Taken together, these results confirmed that induced and accumulated Aβ can be removed after the induction process being terminated in Aβ42 flies.

**Figure 2.**
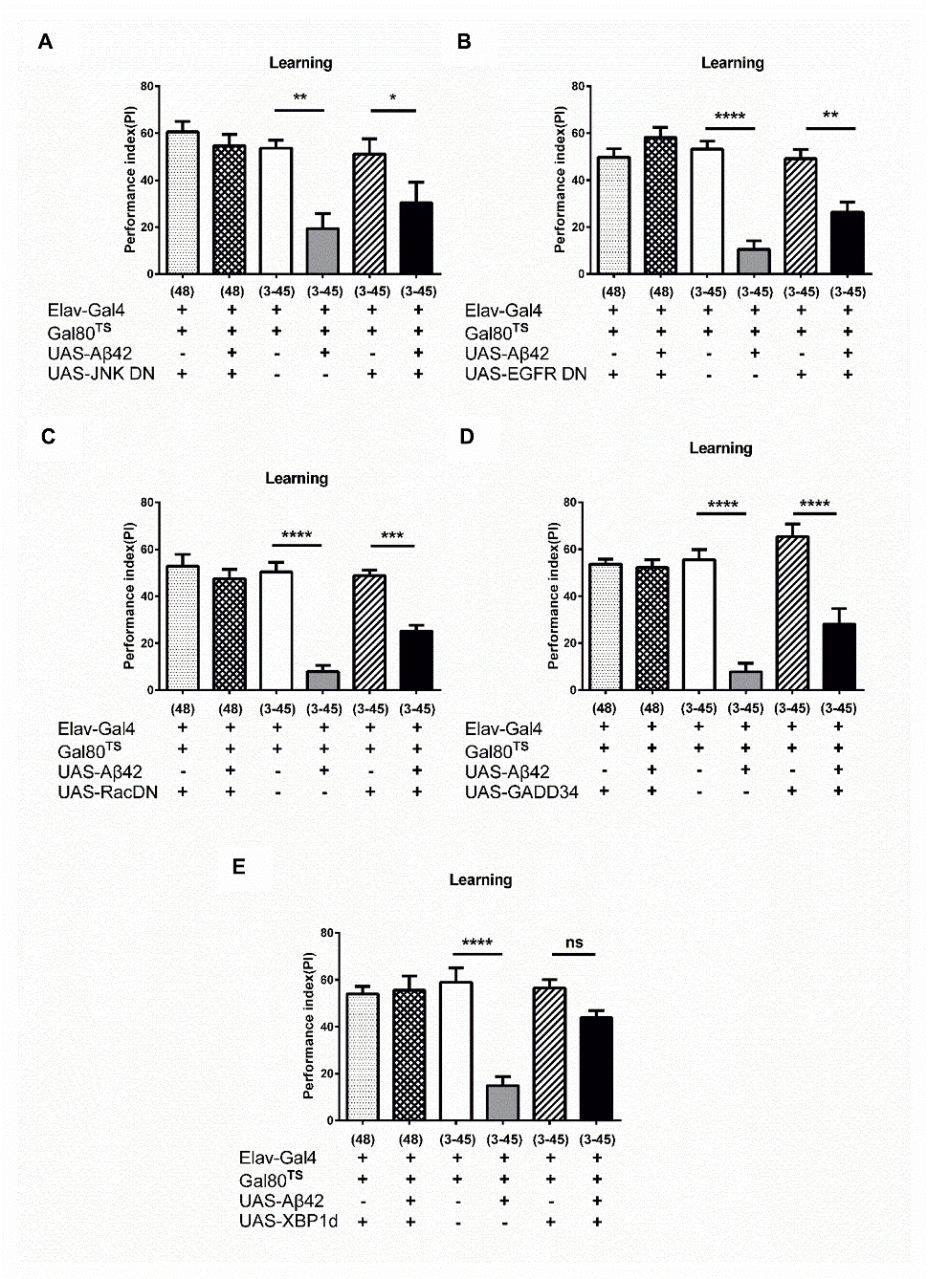
Early XBP1 overexpression prevents the early-transient Aβ accumulation induced learning impairment in later life. (A) Early JNK DN overexpression did not prevent the learning impairment developed in 3_30_°_C_ -45_18_°_C_ Aβ flies (Ctrl (3-45) vs. Aβ42 (3-45): *p* = 0.0043 respectively; Aβ42 (3-45) vs. Aβ42+JNK DN (3-45): *p* = 0.7867, N = 6 respectively). ** *p* < 0.01. (B) Learning impairment was partially improved by early EGFR DN overexpression in 3_30_°_C_ -45_18_°_C_ Aβ flies (Ctrl (3-45) vs. Aβ42 (3-45): *p* < 0.0001 respectively; Ctrl+EGFR DN (3-45) vs. Aβ42+EFDR DN (3-45): *p* = 0.0012 respectively; Aβ42 (3-45) vs. Aβ42+EGFR DN (3-45): *p* = 0.0030, N = 6 respectively). ** *p* < 0.01, **** *p* < 0.0001. (C) Learning impairment was partially improved by Rac DN overexpression in 3_30_°_C_ -45_18_°_C_ Aβ flies (Ctrl (3-45) vs. Aβ42 (3-45): *p* < 0.0001 respectively; Ctrl+Rac DN (3-45) vs. Aβ42+Rac DN (3-45): *p* = 0.0007 respectively; Aβ42 (3-45) vs. Aβ42+Rac DN (3-45): *p* = 0.0197, N = 6 respectively). *** *p*<0.001, **** *p* < 0.0001. (D) Learning impairment was partially improved by GADD34 overexpression in 3_30_°_C_ -45_18_°_C_ Aβ flies (Ctrl (3-45) vs. Aβ42 (3-45): *p* < 0.0001 respectively; GADD34 (3-45) vs. Aβ42+GADD34 (3-45): *p* < 0.0001 respectively; Aβ42 (3-45) vs. Aβ42+ GADD34 (3-45): *p* = 0.0349, N = 6 respectively). * *p* < 0.05, **** *p* < 0.0001. (E) Learning impairment was fully improved by XBP1 overexpression in 3_30_°_C_ -45_18_°_C_ Aβ flies (Ctrl (3-45) vs. Aβ42 (3-45): *p* < 0.0001 respectively; Ctrl+XBP1 (3-45) vs. Aβ42+XBP1 (3-45): *p* = 0.3710 respectively; Aβ42 (3-45) vs. Aβ42+XBP1 (3-45): *p* = 0.0010, N = 6 respectively). ** *p* < 0.01, **** *p* < 0.0001. ns represents non-significant. All Ctrl represents Elav-Gal4+Gal80^TS^.

The memory performance of 3_30_°_C_-21_18_°_C_ Aβ42 flies was examined. As shown in Fig. 1D (P = 0.0020, unpaired Two-tailed T-test) and 1e (P = 0.6168, unpaired Two-tailed T-test), Aβ expression-induced memory damage in 3_30_°_C_ Aβ42 flies was recovered in 3_30_°_C_-21_18_°_C_ Aβ flies. We also examined the memory performance of 3_30_°_C_-3_18_°_C_ Aβ42 flies. Aβ42 flies kept in the 18°C after 3 days in the 30°C displayed damaged memory performance (Supplementary Fig. 2; P = 0.0001, unpaired Two-tailed T-test). The results confirmed that the recovery effect of memory damage in 3_30_°_C_-21_18_°_C_ Aβ flies was due to Aβ removal. However, the recovery of behavioral performance was only temporary in the flies. Despite 3_30_°_C_-30_18_°_C_ Aβ42 flies (*i.e.* flies being housed under the permissive temperature for 30 days after the termination of Aβ induction) did not shown any learning difficulties (Supplementary Fig. 3; P = 0.6066, F (3, 12) = 0.6350, One-way ANOVA), 3_30_°_C_-45_18_°_C_ Aβ42 flies (*i.e.* flies being housed under the permissive temperature for 45 days after the termination of Aβ induction) exhibited noticeable learning deficit (Fig. 1F; P = 0.4877, F (3, 15) = 0.9324, One-way ANOVA). However, the pain sensation and olfactory function of 3_30_°_C_-45_18_°_C_ Aβ42 flies were still retained (Supplementary Fig. 4; Left: P = 0.1622, unpaired Two-tailed T-test; Right: P = 0.6257, unpaired Two-tailed T-test). These results suggest that although the accumulated Aβ in the brain of fly can be cleared after the induction of Aβ being terminated, memory recovery is only temporary and behavioral impairment can be reoccurred in later life.

### Early induction of XBP1 prevents later life learning deficit

To identify molecules or pathways that are responsible for the development of learning deficit in later life, we examined the importance of various molecules that had been shown to be participated in the Aβ-induced learning and memory damage in the past. Increased inflammatory response in the brain has been suggested as one of the major factors affecting neuronal functions and behavioral performance in AD patients [33]. Here, although 8 days overexpression of the dominant-negative K53R c-Jun N-terminal kinase (JNK DN) recovered Aβ-induced learning deficit in Aβ flies (*i.e.* 8_30_°_C_ Aβ flies, 3 days overexpression of JNK DN did not recover the Aβ-induced learning deficit in Aβ flies *(i.e.* 3_30_°_C_-45_18_°_C_ Aβ42 flies) (Supplementary Fig. 5; P =0.0004, F (3, 23) = 9.016, One-way ANOVA and Fig. 2A; P =0.0001, F (5, 30) = 72.82, One-way ANOVA). Previous studies showed that downregulation of EGFR and its downstream signaling pathway reverses learning and memory deficit in Aβ flies [27, 28]. However, in the current study, early overexpression of the dominant-negative EGFR (EGFR DN) and Rac1dominant-negative mutant, N17, Rac DN did not show any prominent recovery effect in 3_30_°_C_-45_18_°_C_ Aβ42 flies (Fig. 2B; P < 0.0001, F (5, 30) = 22.80, One-way ANOVA and 2C; P < 0.0001, F (5, 30) = 25.93, One-way ANOVA). ER stress has been suggested to play an important role in mediating Aβ-induced pathology [16]. Antagonizing the PERK pathway by overexpression GADD34 has been shown to recover Aβ-induced learning deficit [16]. Here, although the ability of learning was improved after early expression of GADD34 in 3_30_°_C_-45_18_°_C_ Aβ42 flies, the performance recovery level was not as good as that of the control flies (Fig. 2D; P < 0.0001, F (5, 24) = 23.57, One-way ANOVA). XBP1 has been shown to play an important role in maintaining the neuronal cell viability of Aβ flies [15, 16]. Here, we found the learning performance was recovered in 3_30_°_C_-45_18_°_C_ Aβ42 (Fig. 2E; P < 0.0001, F (5, 30) = 28.05, One-way ANOVA). Altogether, these behavioral results suggest that the Aβ42-induced memory impairment is reversible, and the molecular mechanism involved in 3_30_°_C_-45_18_°_C_ Aβ42-induced learning impairment is different from the learning damage induced by the continuous Aβ accumulation.

### Increased oxidative stress in 3_30_°_C_-45_18_°_C_ Aβ flies induces learning impairment

**Figure 3.**
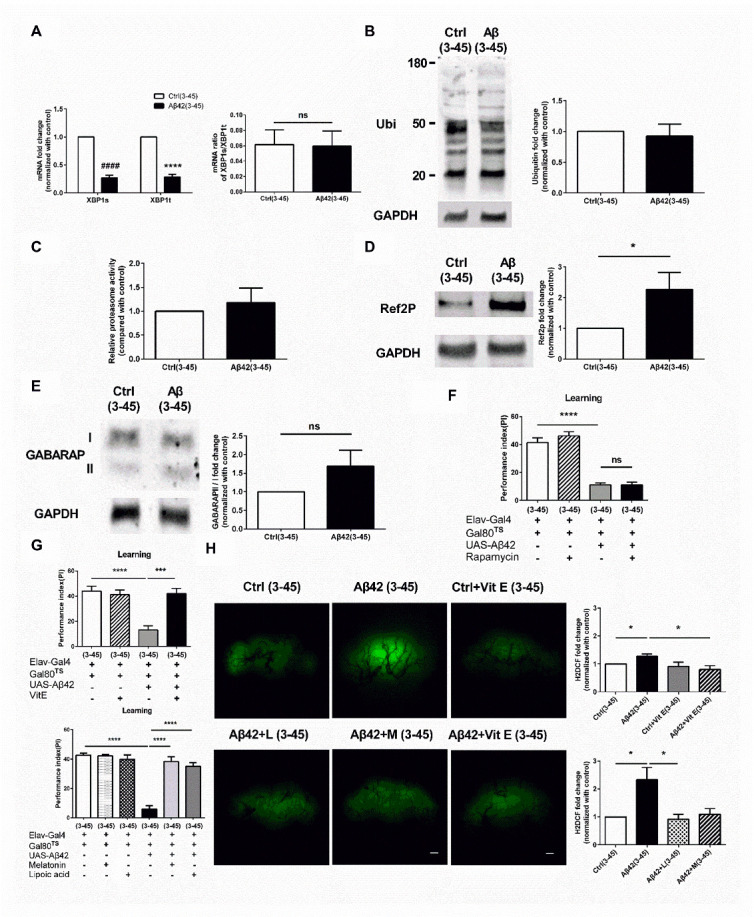
Dysregulation of the oxidative stress response causes learning impairment in 3_30_°_C_ -45_18_°_C_ Aβ flies. (A) Both XBP1t and XBP1s mRNA levels were reduced in the brain of 3_30_°_C_ -45_18_°_C_ Aβ flies, but the ratio of XBP1s/XBP1t remained similar, N = 3 respectively. ****p <0.0001 in XBP1t, ^####^ *p*<0.0001 in XBP1s. (B) The level of protein ubiquitination was not changed in the brain of 3_30_°_C_ -45_18_°_C_ Aβ flies. The quantitative data is shown on the right, N = 7 respectively. (C) The proteasome activity was not changed in brain of 3_30_°_C_ -45_18_°_C_ Aβ flies, N = 3 respectively. (D) The amount of Ref2P was increased in the brain of 3_30_°_C_ -45_18_°_C_ Aβ flies. The quantitative data is shown on the right, N = 12 respectively. *p< 0.05. (E) The GABARAP II/I ratio was not changed in the brain of 3_30_°_C_ -45_18_°_C_ Aβ flies. The quantitative data is shown on the right, N = 15 respectively. (F) Rapamycin treatment for 4 weeks could not improve the learning impairment in 3_30_°_C_ -45_18_°_C_ Aβ flies. (Ctrl (3-45) vs. Aβ42 (3-45): *p*<0.0001 respectively; Aβ42 (3-45) vs. Aβ42+Rapamycin (3-45): *p* > 0.9999, N = 6 respectively). **** *p* < 0.0001. (G) Vitamin E, melatonin (M) or lipoic acid (L) treatment for 4 weeks improved the learning impairment in 3_30_°_C_ -45_18_°_C_ Aβ flies. (Left: Ctrl (3-45) vs. Aβ42 (3-45): *p* < 0.0001; Aβ42 (3-45) vs. Aβ42+Vit. E (3-45): *p* = 0.0002, respectively, *n* = 6; Right: Ctrl (3-45) vs. Aβ42 (3-45): *p* < 0.0001; Aβ42 (3-45) vs. Aβ42+L (3-45): *p* < 0.0001; Aβ42 (3-45) vs. Aβ42+M (3-45): *p* < 0.0001, N = 6 respectively). *** *p* < 0.001, **** *p* < 0.0001. (H) Vitamin E, melatonin (M) or lipoic acid (L) treatment for 4 weeks decreased the levels of ROS in 3_30_°_C_ -45_18_°_C_ Aβ flies. The quantitative data is shown on the right (Right Top: Ctrl (3-45) vs. Aβ42 (3-45): *p* = 0.0270; Aβ42 (3-45) vs. Aβ42+Vit. E (3-45): *p* = 0.0438; Right bottom: Ctrl (3-45) vs. Aβ42 (3-45): *p* = 0.0389; Aβ42 (3-45) vs. Aβ42+L (3-45): *p* = 0.0201; Aβ42 (3-45) vs. Aβ42+M (3-45): *p* = 0.0391, N = 3 respectively). * *p* < 0.05. ns represents non-significant. All Ctrl represents Elav-Gal4+Gal80^TS^. White bar indicates 50 μm.

To determine the XBP1 expression level in 3_30_°_C_-45_18_°_C_ Aβ flies, quantitative PCR was used. The results showed that expression of both the total and the splicing form of XBP1 was reduced in 3_30_°_C_-45_18_°_C_ Aβ flies, as compared to the age-matched control flies (Fig. 3A; Left: *XBP1s*: P < 0.0001, unpaired Two-tailed T-test; *XBP1t*: P < 0.0001, unpaired Two-tailed T-test; Right: P = 0.9524, unpaired Two-tailed T-test). Although the reduction of PERK and ATF6 was also found in 3_30_°_C_-45_18_°_C_ Aβ flies (Supplementary Fig. 6; PEK: P < 0.0001, unpaired Two-tailed T-test; CRC: P < 0.0001, unpaired Two-tailed T-test; ATF6: P < 0.0001, unpaired Two-tailed T-test); however, considering the behavioral results as shown in Fig. 2D and E, we believed that reduction of XBP1 is one of the major causes of the induction of learning impairment in 3_30_°_C_-45_18_°_C_ Aβ flies. XBP1 regulates numerous cellular functions such as proteasome activity, autophagy and oxidative stress [18, 34, 35]. Western blot analysis was used to examine the protein ubiquitination and autophagy level in the brain of 3_30_°_C_-45_18_°_C_ Aβ flies. As shown in Fig. 3B (P = 0.7148, unpaired Two-tailed T-test), the overall protein ubiquitination level remained similar in the brain of 3_30_°_C_-45_18_°_C_ Aβ flies, as compared to the control flies. We also used proteasome activity assay to confirm that the proteasome activity of 3_30_°_C_-45_18_°_C_ Aβ flies remained similar to the control flies (Fig. 3C; P = 0.5958, unpaired Two-tailed T-test). Despite increased of Ref(2)p, a homolog of mammalian p62/SQSTM1, was found in the brain of 3_30_°_C_-45_18_°_C_ Aβ flies, the expression ratio of GABARAP II/I remained similar compared to the control flies (Fig. 3C; P = 0.0336, unpaired Two-tailed T-test and 3d; P = 0.1141, unpaired Two-tailed T-test). In addition, feeding rapamycin, which is a widely used autophagy inducer, for 4 weeks did not affect the learning performance of 3_30_°_C_-45_18_°_C_ Aβ flies (Fig. 3E; P < 0.0001, F (3, 20) = 54.22, One-way ANOVA). Co-induction of Drosophila FOXO (dFOXO) with Aβ for 3 days did not affect the learning performance of 3_30_°_C_-45_18_°_C_ Aβ flies (Supplementary Fig. 7; P < 0.0001, F (5, 30) = 28.05, One-way ANOVA). These results suggest that autophagy is not involved in the development of learning deficit of 3_30_°_C_-45_18_°_C_ Aβ flies. To examine the role of ROS in 3_30_°_C_-45_18_°_C_ Aβ flies, flies were fed with three different types of antioxidants, vitamin E, melatonin (M) or lipoic acid (L), for 4 weeks prior to the behavioral analysis. Results showed that antioxidants treatment prevented the development of learning deficit in 3_30_°_C_-45_18_°_C_ Aβ flies (Fig. 3F; Left: P < 0.0001, F (3, 20) = 15.08; Right: P < 0.0001, F (5, 30) = 33.87, One-way ANOVA). 2',7'-dichlorodihydro-fluorescein (H2DCF, an indicator for ROS) staining further confirmed that 3_30_°_C_-45_18_°_C_ Aβ flies have higher intracellular ROS accumulation than to the control flies (Fig. 3G; Right Top: P = 0.0827, F (3, 8) = 3.218; Right bottom: P = 0.0146, F (3, 8) = 6.632, One-way ANOVA). These results suggest that increased ROS level plays an important role in the development of learning deficit in 3_30_°_C_-45_18_°_C_ Aβ flies.

**Figure 4.**
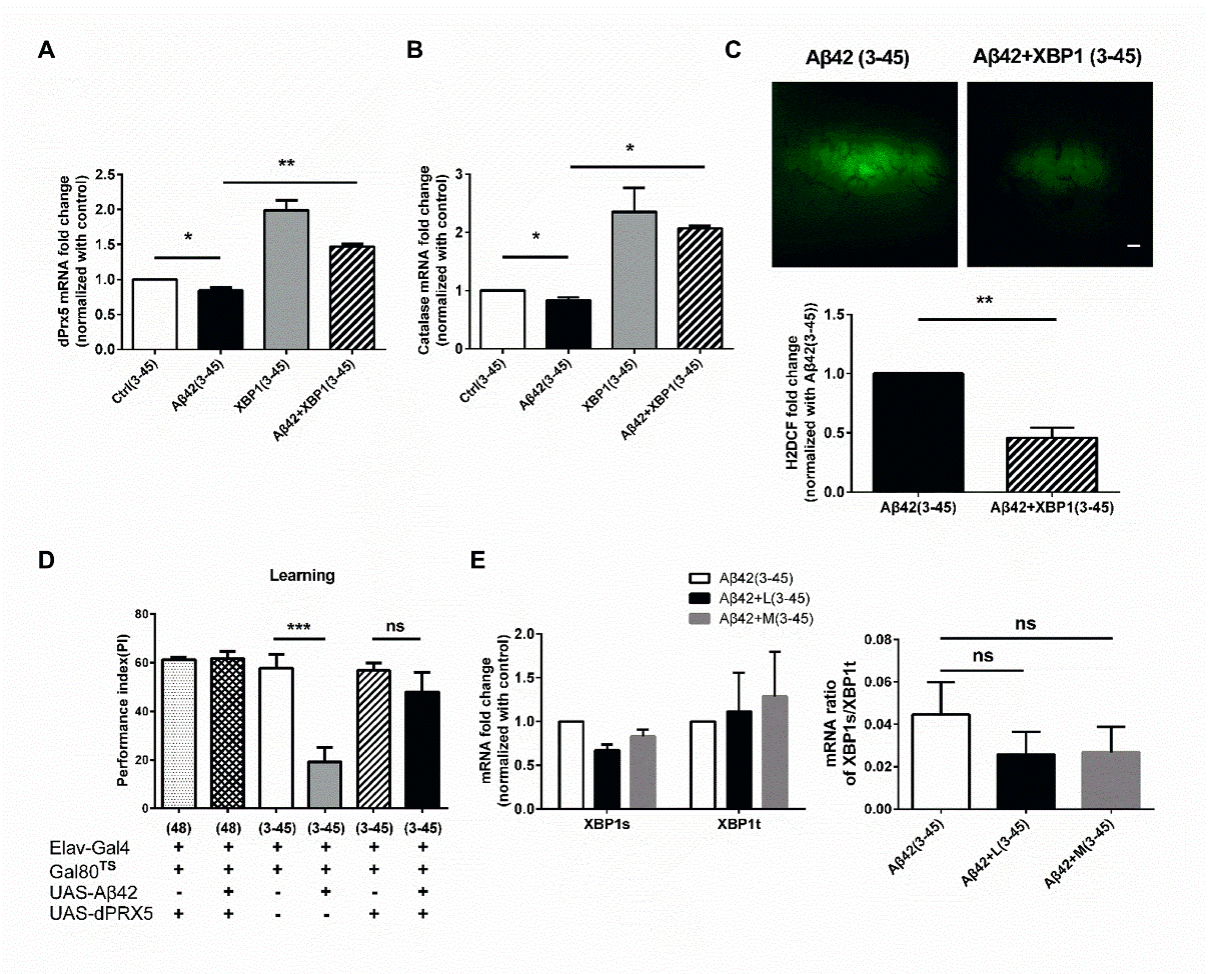
Early XBP1 expression increases the expression of catalase and dPrx5, reduces oxidative stress, and improves learning performance in 3_30_°_C_ -45_18_°_C_ Aβ flies. (A) The reduction of *dPrx5* mRNA in 3_30_°_C_ -45_18_°_C_ Aβ flies was prevented by early XBP1 overexpression (Ctrl (3-45) vs. Aβ42 (3-45): *p* = 0.0483; Aβ42 (3-45) vs. Aβ42+XBP1 (3-45): *p* = 0.0017, N = 3 respectively). * *p* < 0.05, ** *p* < 0.01. (B) The reduction of *catalase* mRNA in 3_30_°_C_ -45_18_°_C_ Aβ flies was prevented by early XBP1 overexpression (Ctrl (3-45) vs. Aβ42 (3-45): *p* = 0.0476; Aβ42 (3-45) vs. Aβ42+XBP1 (3-45): *p* = 0.0135, N = 3 respectively). *p< 0.05. (C) Early XBP1 overexpression for 3 days prevented the oxidative stress elevation in 3_30_°_C_ -45_18_°_C_ Aβ flies. The quantitative data is shown at the bottom, N = 3 respectively. ** *p* < 0.01. ns represents non-significant. (D) Learning impairment in 3_30_°_C_ -45_18_°_C_ Aβ flies was fully improved by early dPrx5 overexpression. (Ctrl (3-45) vs. Aβ42 (3-45): *p* = 0.0002; Aβ42 (3-45) vs. Aβ42+dPrx5 (3-45): *p* = 0.0057; Ctrl (3-45) vs. Aβ42+dPrx5 (3-45): *p* = 0.7417, N = 6 respectively). ** *p* < 0.01, *** *p* < 0.001. (E) The XBP1s and XBP1t mRNA expression level and the ratio of XBP1s/XBP1t did not change in the brain of 3_30_°_C_ -45_18_°_C_ Aβ flies with melatonin (M) or lipoic acid (L) co-treatment (Left: XBP1t: Aβ42 (3-45) vs. Aβ42+L (3-45): *p* = 0.9889; Aβ42 (3-45) vs. Aβ42+M (3-45): *p* = 0.8599; XBP1s: Aβ42 (3-45) vs. Aβ42+L (3-45): *p* = 0.8040; Aβ42 (3-45) vs. Aβ42+M (3-45): *p* = 0.9639; Right: Aβ42 (3-45) vs. Aβ42+L (3-45): *p* = 0.6928; Aβ42 (3-45) vs. Aβ42+M (3-45): *p* = 0.7274, N = 3 respectively). All Ctrl represents Elav-Gal4+Gal80^TS^. White bar indicates 50 μm.

To dissect the mechanisms underlying ROS upregulation in 3_30_°_C_-45_18_°_C_ Aβ flies, we examined the gene expression of various antioxidants. Results of the qPCR analysis showed that expression of dPrx5 and catalase was decreased in 3_30_°_C_-45_18_°_C_ Aβ flies (Fig. 4A; P < 0.0001, F (3, 8) = 45.81, One-way ANOVA and 4b; P < 0.0001, F (3, 8) = 45.81, One-way ANOVA). In addition, co-induction of XBP1 and Aβ for 3 days increased the expression of dPrx5 and catalase in 3_30_°_C_-45_18_°_C_ Aβ flies, suggesting that early induction of XBP1 might prevent antioxidant reduction in later life. This hypothesis was supported by results showing that 3 days co-expression of XBP1 with Aβ reduced the ROS accumulation in 3_30_°_C_-45_18_°_C_ Aβ flies (Fig. 4C; P = 0.0033, unpaired Two-tailed T-test). Accumulating evidence suggested that dPrx5 is one of the antioxidants in flies with abundant expression in the brain [36]. Behavioral study confirmed that 3 days co-expression of dPrx5 with Aβ prevented the occurrence of learning damage in 3_30_°_C_-45_18_°_C_ Aβ flies (Fig. 4D; P < 0.0001, F (5, 24) = 10.38, One-way ANOVA). Further study showed that antioxidants, melatonin or lipoic acid, treatment did not increase the XBP1 expression in 3_30_°_C_-45_18_°_C_ Aβ flies, suggesting that decreased XBP1 expression cannot be reversed by antioxidant treatment (Fig. 4E; Left: *XBP1t*: P = 0.8404, F (2, 8) = 0.1756; *XBP1s*: P = 0.8404, F (2, 6) = 0.1756; Right: P = 0.5292, F (2, 9) = 0.6836, One-way ANOVA).

**Figure 5.**
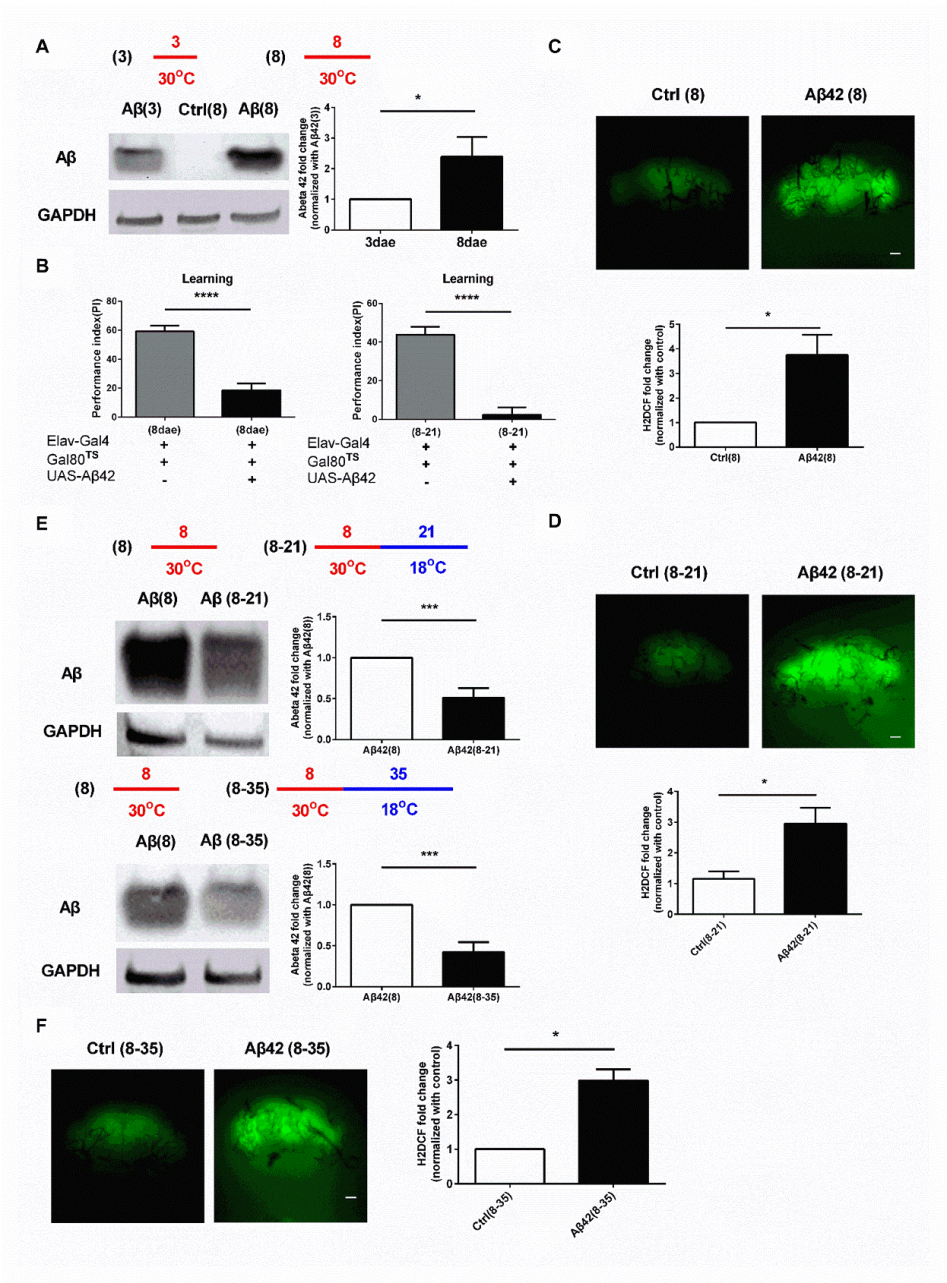
Prolonged Aβ induction causes learning impairment that cannot be reversed by reducing Aβ accumulation. (A) The level of Aβ accumulation in flies with 8 days induction was higher than that with 3 days induction, N = 3 respectively. * *p* < 0.05. (B) The learning impairment occurred in 8_30_°_C_ Aβ flies was not improved in 8_30_°_C_-21_18_°_C_ Aβ flies, N = 6 respectively. **** *p* < 0.0001. (C) Increased ROS level was found in 8_30_°_C_ flies. The quantitative data is shown at the bottom, N = 4 respectively. * *p* < 0.05. (D) Increased ROS level was also found in 8_30_°_C_-21_18_°_C_ Aβ flies. The quantitative data is shown on the right, N = 3 respectively. * *p* < 0.05. (E) The Aβ level in 8_30_°_C_-21_18_°_C_ and 8_30_°_C_ -35_18_°_C_ Aβ flies was lowered by approximately 50% as compared to that in 8_30_°_C_ flies. The quantitative data is shown on the right, N = 6 respectively. ***p < 0.001. (F) The ROS level in 8_30_°_C_ -35_18_°_C_ Aβ flies was higher than that in the control flies. The quantitative data is shown at the bottom, N = 3 respectively. * *p* < 0.05. All Ctrl represents Elav-Gal4+Gal80^TS^. White bar indicates 50 μm.

### Prolonged Aβ induction-induced learning impairment and oxidative stress cannot be reversed by reducing Aβ accumulation

To understand if the prolonged Aβ induction-induced learning damage is reversible, we performed experiments with an extended Aβ induction period. Prolonged induction of Aβ induces learning deficit in flies [16, 27]. Here, flies with 8 days Aβ induction (*i.e.*, 8_30_°_C_ Aβ flies) showed higher Aβ accumulation in the brain than flies with 3 days Aβ induction, as expected (Fig. 5A; P = 0.0166, unpaired Two-tailed T-test). However, similar levels of learning deficit were still observed in and between flies with prolonged Aβ induction (*i.e.*, 8_30_°_C_ Aβ flies) only and those with the removal of Aβ after prolonged induction (8_30_°_C_-21_18_°_C_ Aβ flies) (Fig. 5B; Right: P < 0.0001, unpaired Two-tailed T-test; Left: P < 0.0001, unpaired Two-tailed T-test). Results of the H2DCF staining revealed that the ROS levels remained high in both 8_30_°_C_ Aβ flies and 8_30_°_C_-21_18_°_C_ Aβ flies (Fig. 5C; P = 0.0161, unpaired Two-tailed T-test and 5d; P = 0.0345, unpaired Two-tailed T-test), suggesting that continuous accumulation of ROS (or fail to clear ROS) could play a role in learning damage in 8_30_°_C_-21_18_°_C_ Aβ flies. Further examination in 8_30_°_C_-35_18_°_C_ Aβ flies showed less than 10% of the accumulated Aβ was further reduced (the rest amount of Aβ in 8_30_°_C_-35_18_°_C_ Aβ flies was 0.42±0.15 v.s. 0.51±0.17 in in 8_30_°_C_-21_18_°_C_ Aβ flies) and the levels of ROS of 8_30_°_C_-35_18_°_C_ Aβ flies remain similar to 8_30_°_C_-21_18_°_C_ Aβ flies (Fig. 5E; Top: P = 0.0010, unpaired Two-tailed T-test; Bottom: P = 0.0009, unpaired Two-tailed T-test and 5f; P = 0.0273, unpaired Two-tailed T-test). These results suggest that longer Aβ exposure might both increase Aβ accumulation to a level that cannot be fully cleared endogenously and induce ROS accumulation to a level that exceed the maximal ROS removal capacity of cells, leading to neuronal damage and learning impairment.

### Early Aβ exposure makes cell more vulnerable to the oxidative stress in later life

**Figure 6.**
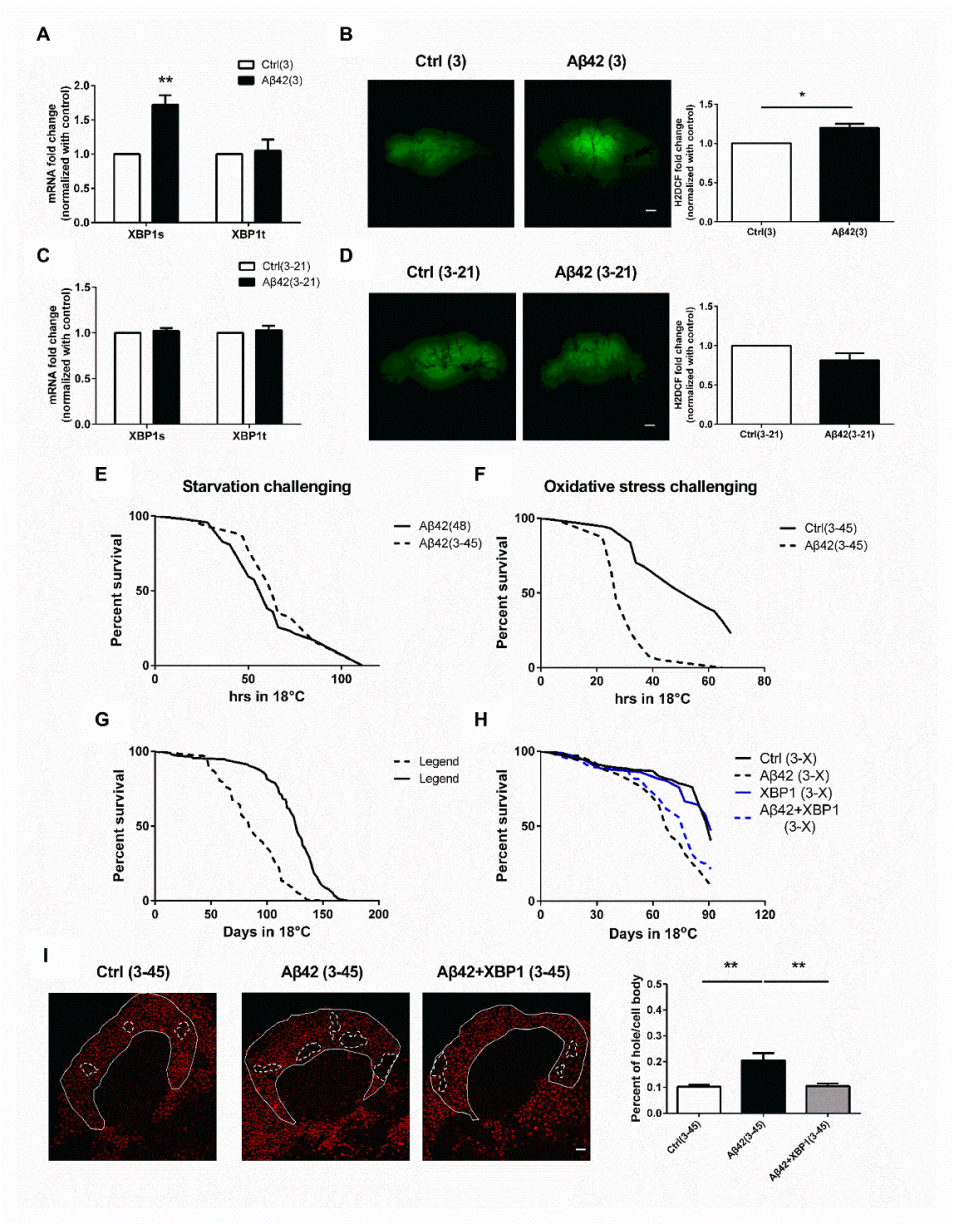
Early XBP1 expression improves the resilience to oxidative stress and prevents cells death in later life. (A) The XBP1s, but not XBP1t, mRNA level was increased in the brain of 3_30_°_C_ Aβ flies, N = 3 respectively. **p < 0.01. (B) Increased ROS level was found in 3_30_°_C_ Aβ flies. The quantitative data is shown at the bottom, N = 4 respectively. * *p* < 0.05. White bar indicates 50 μm. (C) Both XBP1t and XBP1s mRNA level had not been significantly changed in the brain of 3_30_°_C_ -21_18_°_C_ Aβ flies, N = 3 respectively. (D) The ROS in 3_30_°_C_ -21_18_°_C_ Aβ flies was similar to control flies. The quantitative data is shown at the bottom, N = 8 respectively. ***p < 0.001. White bar indicates 50 μm. (E) The survival rate of 3_30_°_C_ -45_18_°_C_ Aβ flies was similar to that of the age matched control (48_18_°_C_) flies upon starvation challenge. *n*= 70-75 flies for each genotype. (F) The survival rate of 3_30_°_C_ -45_18_°_C_ Aβ flies was reduced after paraquet treatment. *n*= 70-75 flies for each genotype. (G) Early Aβ accumulation induced early death. Both the Aβ and the control group were housed under 30°C for 3 days after eclosion and then transferred to 18°C for life-span analysis. *n*= 110-120 flies for each genotype. (3-X) represents (3_30_°_C_ -X_18_°_C_) (H) Early XBP1 overexpression improved the life-span. *n*= 110-120 flies for each genotype. (I) Early XBP1 overexpression prevented cell loss induced by early Aβ expression in the brain, as examined by the propidium iodide staining (*F*_(2, 12)_ = 11.05, *p* = 0.0019; Ctrl (3-45) vs. Aβ42 (3-45): *p* = 0.0039, respectively; Aβ42 (3-45) vs. Aβ42+XBP1 (3-45): *p* = 0.0043, respectively, n = 5). Areas around calyx were quantified and the quantification was done under the double-blind procedure. White circles represent the vacuolated area. The quantitative results are shown on the right. White bar indicates 5 μm. **p < 0.01. All Ctrl represents Elav-Gal4+Gal80^TS^.

As we found that early Aβ accumulation decreases XBP1 expression in 3_30_°_C_-45_18_°_C_ Aβ flies, we hypothesized that reducing the expression of catalase and dPrx5 might make cells more vulnerable to the oxidative stress. Here, we sought to understand the XBP1 expression pattern and determine the ROS accumulation in different stage of Aβ flies. Overexpression of Aβ increased the expression of the spliced XBP1 (XBP1s) in 3_30_°_C_ Aβ flies (Fig. 6A; XBP1s: P = 0.0061, unpaired Two-tailed T-test; XBP1t: P = 0.9859, unpaired Two-tailed T-test), suggesting early Aβ accumulation promotes ER stress response. Results of the confocal microscopy showed that the levels of ROS were higher in 3_30_°_C_ Aβ flies than to control flies (Fig. 6B; P = 0.0161, unpaired Two-tailed T-test). This result suggests that the presence of Aβ increases oxidative stress and causes ER stress activation to promote XBP1 splicing. This result was consistent with our previous report [16]. Interestingly, the expression of XBP1s was decreased back to the basal level and the levels of ROS in 3_30_°_C_-21_18_°_C_ Aβ flies were similar to that in the control flies (Fig. 6C; XBP1s: P = 0.9481, unpaired Two-tailed T-test; XBP1t: P = 0.8721, unpaired Two-tailed T-test and 6d; P = 0.0010, unpaired Two-tailed T-test). However, the levels of ROS in 3_30_°_C_-45_18_°_C_ Aβ flies were higher than that in control flies (Fig. 3G). Therefore, we hypothesized that decreased XBP1 and antioxidants in the aged Aβ flies make animal less resistant to the ROS stress. To confirm our hypothesis, we challenged the flies with two different conditions, starvation and paraquet challenge. Results from the starvation challenge experiment showed that the survival rate of 3_30_°_C_-45_18_°_C_ Aβ flies was similar to the age-matched control flies (Fig. 6e; P = 0.7994, Log-rank test). Intriguingly, paraquet feeding reduced the life-span of 3_30_°_C_-45_18_°_C_ Aβ flies, as compared to the control group (Fig. 6F; P < 0.0001, Log-rank test). These results confirmed that 3_30_°_C_-45_18_°_C_ Aβ flies are highly vulnerable to the oxidative stress but not to the starvation stress. In addition, results of the H2DCF staining showed that early induction of XBP1 reduced the accumulation of ROS in 3_30_°_C_-45_18_°_C_ Aβ flies (Fig. 4D), further supporting that excessive ROS is one of the main factors to trigger the development of the pathological behaviors. Longevity assay showed that not only the learning performance was impaired in the early Aβ induced flies, but also the life-span. As shown in Fig. 6G (P < 0.0001, Log-rank test), early Aβ exposure caused life-span shortening in flies. Our previous study showed that overexpression of XBP1 extends the life-span of Aβ flies [16]. To understand the role of XBP1 in early Aβ exposure-induced pathology, the life-span and cell viability were examined. Our results showed that early induction of XBP1 extended the life span of the early Aβ exposed flies (Fig. 6H; Ctrl vs. Aβ42: P < 0.0001; Aβ42 vs. Aβ42+XBP1: P = 0.0119, Log-rank test) and improved the cell viability in 3_30_°_C_-45_18_°_C_ Aβ flies. The vacuolated area in the cell body region of mushroom body was quantified and examined by propidium iodide staining (Fig. 6I; P = 0.0019, F (2, 12) = 11.05, One-way ANOVA).

## DISCUSSION

The current study aims to understand the molecular-pathological changes occurred after the clearance of Aβ accumulation and to determine the role of these changes in the development of learning impairment in later life. Findings of the current study reveal that an endogenous Aβ clearance system is present in flies for the removal of Aβ. However, the reduction of Aβ accumulation only temporary recovers the memory deficit. At the molecular level, early Aβ exposure reduces the expression of XBP1, catalase and dPrx5 in later life and affects animal stress response. This study, for the first time, confirms that early-transient Aβ exposure, even being removed soon afterwards, has a pathological impact on the animal’s life.

### Existence of an endogenous Aβ degradation system in fruit fly

The clearance of Aβ in the brain can be carried out through two different mechanisms - the “active” and the “passive” clearance pathways. Aβ is actively removed by degrading enzymes, cellular degradation systems, or be taken up by glia cells. Degrading enzymes including neprilysin and insulin-degrading enzyme (IDE) has been demonstrated to play an important role in this active protein removal process in flies [37, 38]. Cellular degradation systems have also been shown to degrade Aβ in the fruit fly [39]. Glia cells in the brain of flies provide a protection to neurons from Aβ toxicity [40]. On the other hand, the passive mechanism includes diffusion of Aβ from brain to the peripheral [41, 42]. In the current study, we did not identify which mechanism is responsible for the clearance of Aβ and the recovery of the pathological behaviors. It would be of great interest to identify the major Aβ degradation/removal system in flies in the future.

### The effects of transient and prolonged Aβ exposure on cell’s functions are different

Despite Aβ peptides are degradable, endogenously, at different stage; the result of behavioral recovery is different. Memory damage was recovered in 3_30_°_C_-21_18_°_C_ Aβ flies, while learning deficit was not reversed in 8_30_°_C_-21_18_°_C_ Aβ flies. Why the learning damage cannot be recovered? Multiple mechanisms underlying this phenomenon can potentially be existed and two of the possible mechanisms are: 1) Impairment of the endogenous Aβ degradation system. The amount of Aβ left in 8_30_°_C_-21_18_°_C_ Aβ flies could not be further degraded or removed as in 8_30_°_C_-35_18_°_C_ Aβ flies, suggesting the amount of Aβ accumulated in cells exceeds the capacity of the endogenous Aβ degradation system and 2) Continuous accumulation of ROS. Our experiment results showed that oxidative stress still remained high in 8_30_°_C_-21_18_°_C_ Aβ flies, even though about half of the accumulated Aβ was soon being removed after the prolonged Aβ induction, suggesting the assault of oxidative stress was not ceased. This data suggests that prolonged Aβ exposure and excessive Aβ accumulation would damage cells and these damages cannot be easily recovered endogenously. Therefore, we hypothesize that the quantity (*i.e.*, the amount of Aβ accumulated in cells), and the quality (*i.e.*, the duration of Aβ accumulation) are both important for the development of Aβ-induced toxicity. Our data suggests that short-term Aβ exposure-caused functional changes can be recovered and are reversible if Aβ is soon removed by the endogenous Aβ clearing system but prolonged Aβ exposure-induced cellular functional changes (*e.g.*, degradation system malfunction) and is difficult to recover as the amount of Aβ accumulated is larger and it is more difficult to remove the accumulated Aβ by the endogenous Aβ clearing system.

### Differential mechanisms involved in the post Aβ clearance-induced toxicity and the continuous Aβ accumulation-induced toxicity

Our results revealed that the mechanism involved in the development of pathological behaviors in flies with the removal of Aβ after Aβ accumulation and those with continuous Aβ accumulation is different. Many cellular signaling pathways have been shown to play a role in the abnormal learning and memory behaviors development in flies with continuous Aβ expression. In our current study, manipulating these pathways failed to prevent the development of behavioral damage in early Aβ exposed flies, suggesting another mechanism is presence in mediating the neurological (*i.e.* pathological) changes in later life. Notably, the ROS accumulation-induced learning impairment was found only in the aged animal. The ROS level was increased following Aβ induction (as shown in 3_30_°_C_ Aβ flies), returned to the normal soon after (*i.e.*, 21 days) the initiation of the removal of Aβ (as shown in 3_30_°_C_-21_18_°_C_ Aβ flies), but increased again awhile (*i.e.* 45 days) after the removal of Aβ (as shown in 3_30_°_C_-45_18_°_C_ Aβ flies). This finding suggests that the aging process may also contribute to the development of learning and memory impairment in animals experienced with the early Aβ exposure. Decreased tolerance to the oxidative stress, declined behavioral performance and increased amount of the dysfunctional ER have all been documented as the features of aging [27, 43]. We hypothesize that the ability to resist oxidative stress decreased as animals getting older (*i.e.*, aged), especially for those with cells that had experienced Aβ exposure in early life. In addition, the increasing ROS accumulation during aging further increases the cells’ burden in maintaining the balance of the ROS levels, making cells more difficult in carrying out various physiological processes. Could early Aβ exposure facilitate the aging process? Or early Aβ exposure amplifies the effects of aging? Our results suggest that early Aβ exposure partially amplifies the aging effects, especially on the oxidative stress response. It would be of great interest for further study to reveal the relation between Aβ exposure and the aging process.

### Oxidative stress triggers learning impairment in later life

Oxidative stress has long been suggested involving in the aging process and the development of various diseases including AD. As aging is the major risking factor for AD, the role of ROS in the development of AD and aging has attracted scientist’s attentions [44]. Current study suggests that Aβ exposure induced XBP1 dysregulation disrupts the expression of various oxidative stress response molecules in later life, which decreases cells’ ability to clear ROS and subsequently leads to the induction of behavioral damage.

Activation of XBP1 has been suggested to protect cells from oxidative stress. XBP1-deficient cells show decreased catalase expression and increased ROS production [45]. Moreover, it has been shown that knockdown of XBP1 reduces antioxidant genes expression in the human RPE cells [46]. Accumulated evidence has suggested XBP1 activation is cell’s early response during the development of AD. The XBP1 expression is found transiently increased in the APP/PS1 and 5xFAD mice and also in the human AD cases [17]. In supporting this, findings of the current and other studies using Aβ flies as a model also suggest that XBP1 splicing is a cellular early response to protect cells from Aβ toxicity [15, 16]. Of note, current study further demonstrated that XBP1 dysregulation affects learning performance in later life. Our results showed that early Aβ exposure leads to the reduction of both the total and the spliced forms of XBP1 in 3_30_°_C_-45_18_°_C_ Aβ flies. Ectopic expression of XBP1 during the 3 days Aβ induction period prevents the learning damage in 3_30_°_C_-45_18_°_C_ Aβ flies. Further analysis indicated the involvement of proteasome and autophagy in memory impairment was unlikely in 3_30_°_C_-45_18_°_C_ Aβ flies (Fig. 3). Collectively, findings of this study suggest that oxidative stress is the major factor that contributes to the development of behavioral damage, which is affected by XBP1 expression. 1) Although increased oxidative stress after 3 days of Aβ induction was reduced after Aβ accumulation is reduced in 3_30_°_C_-21_18_°_C_ Aβ flies, increased oxidative stress was observed, again, in 3_30_°_C_-45_18_°_C_ Aβ flies. 2) Co-expression of XBP1 with Aβ for 3 day induction prevents the oxidative stress increase in 3_30_°_C_-45_18_°_C_ Aβ flies. 3) Co-expression of dPrx5 with Aβ for 3 days induction prevents the behavioral damage in 3_30_°_C_-45_18_°_C_ Aβ flies. 4) Reduction of dPrx5 expression in 3_30_°_C_-45_18_°_C_ Aβ flies is prevented if XBP1 is co-expressed with Aβ for 3-day induction. 5) Antioxidant treatment after Aβ clearance can prevent learning damage in 3_30_°_C_-45_18_°_C_ Aβ flies.

Various clinical and pre-clinical AD studies showed that persistent stress decreases XBP1 splicing and ER stress [16, 17]. Here, we propose that transient Aβ exposure is already strong enough to weakening the ER stress response and hampering cell’s ability to neutralize the cellular stress, even after the accumulated Aβ has been removed.

### Maintaining cognitive function after Aβ clearance

It remains unclear on why early TBI experience increases the chance of dementia and neurodegeneration in later life. A recent meta-analysis showed that TBI increases the rate of AD about 1.5 times [47]. A study from military veterans shows that TBI increases the risk of having Parkinson’s disease by approximately 56% [48]. Although a number of studies have demonstrated a transient increase of Aβ accumulation in the region of brain injury [5, 49], the effect of early Aβ accumulation in the late development of neurodegeneration and dementia is largely unknown. Results of the current study confirmed that early-transient Aβ expression/ accumulation promotes the development of learning and memory deficits together with cell death in later life, partially resembling the situations of having TBI in humans. Our data suggest that increased ROS and decreased ER function are two major causes of neuronal dysfunction in later life and the application of antioxidants can prevent the development of learning and memory deficits and cell function in animals with early Aβ exposure. Further our results also propose that the combination of using current developed small molecular to facilitate Aβ clearance and antioxidant treatment would benefit the cognitive function in the AD. Although more studies are needed to further uncover the detailed underlying molecular mechanisms, results of this study suggest that the Aβ fly model is suitable for use to study the effects of post Aβ toxicity.

Decades of studies on Aβ-induced pathologies mainly focus on the effects of the continuous Aβ accumulation. Our study, for the first time, showed the toxic effects of the early-transient Aβ accumulation. We demonstrated that the mechanism underlying its toxic effect is different to that of the continuous Aβ accumulation and further revealed the pathological role of the dysregulated ER stress response and ROS balance induced by the early-transient Aβ accumulation. Previous studies show that reduced Aβ accumulation in the brain does not accompany with cognitive improvement. These observations not only raise a question on the role of Aβ during AD disease progression but also challenge the authentic of Aβ hypothesis. The current results not only link early Aβ accumulation to the post Aβ clearance but also give further evidence for supporting the Aβ hypothesis.

## Supplementary Materials

The Supplementary data can be found online at: www.aginganddisease.org/EN/10.14336/AD.2021.1015.


