# Supplementary material for: Early Aβ42 Exposure Causes Learning Impairment in Later Life
Source: Aging Dis. 2022 Jun 1;13(3):868–83. doi: 10.14336/AD.2021.1015 (PMC9116909; doi:10.14336/AD.2021.1015)
Supplement: Supplementary file 1 [file AD-13-3-868-s.pdf]

SUPPLEMENTARY DATA

Early Aβ42 Exposure Causes Learning Impairment in Later Life

Kuan-Chung Cheng[1,2], Chun Hei Antonio Cheung[1,2], Hsueh-Cheng Chiang[1,2,*]

© 2021. Cheng KC et al. Published online at http://www.aginganddisease.org/EN/10.14336/AD.2021.1015

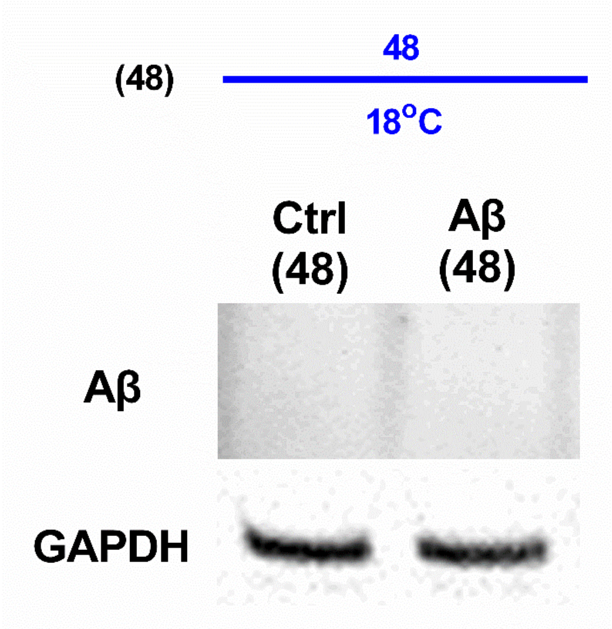

Supplementary Figure 1. No leakage of Aβ expression observed in the permissive temperature. Western blotting data showed that there was no leakage of Aβ expression in the permissive temperature for 48days. $n = 4$ for each group. Ctrl represents Elav-Gal4+Gal80^{TS}.

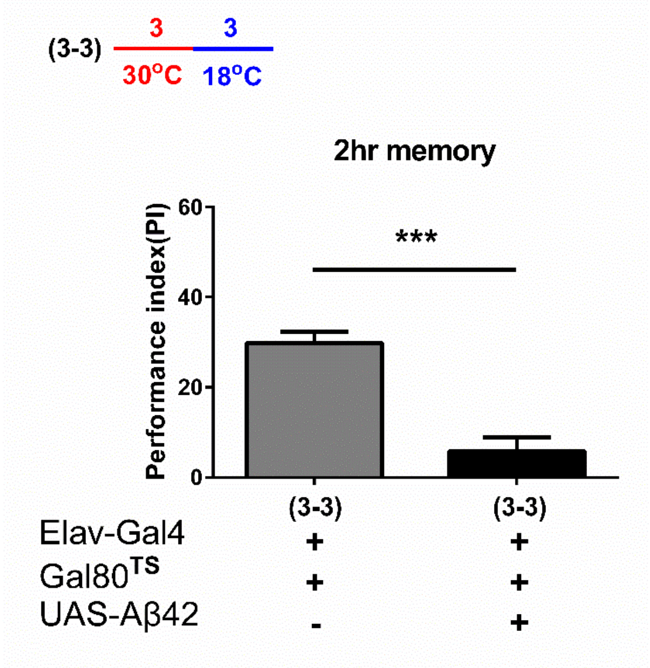

Supplementary Figure 2. The memory damage was occurred in $3_{30°C}$-$3_{18°C}$ Aβ flies. The memory performance was impaired in $3_{30°C}$-$3_{18°C}$ Aβ flies, N = 6 respectively.

© 2021. Cheng KC et al. Published online at http://www.aginganddisease.org/EN/10.14336/AD.2021.1015

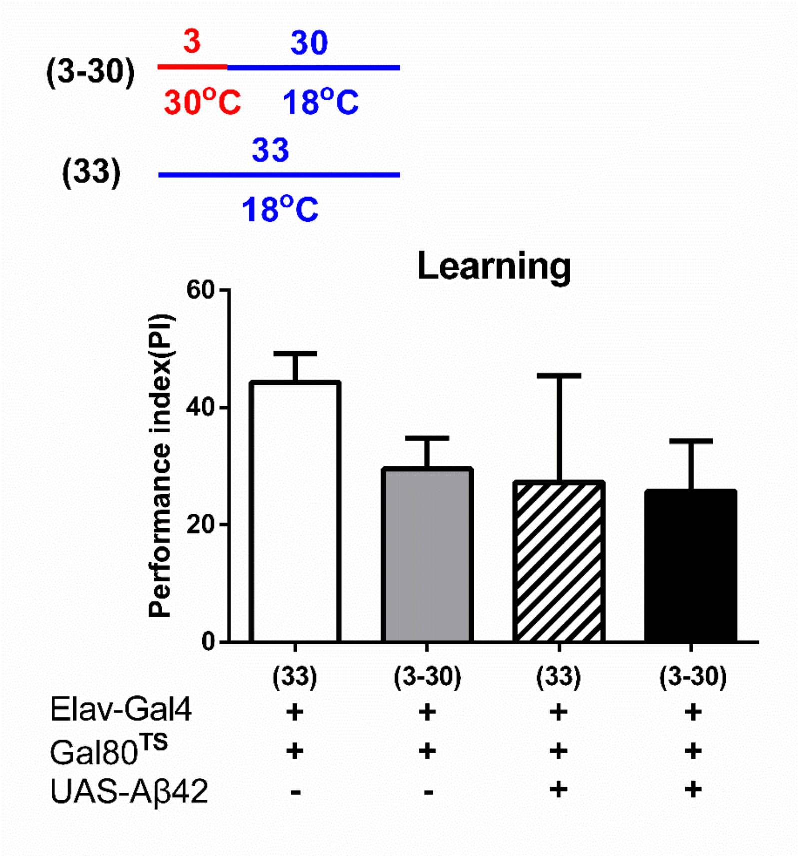

Supplementary Figure 3. The learning impairment was not occurred in $3_{30^{o}C}$ -$30_{18^{o}C}$ Aβ flies. The normal learning ability performed in Aβ group of $3_{30^{o}C}$ -$30_{18^{o}C}$ flies (Ctrl (3-30) vs. Aβ42 (3-30): $p = 0.9943$, N = 6 respectively). All Ctrl represents Elav-Gal4+Gal80^{TS}.

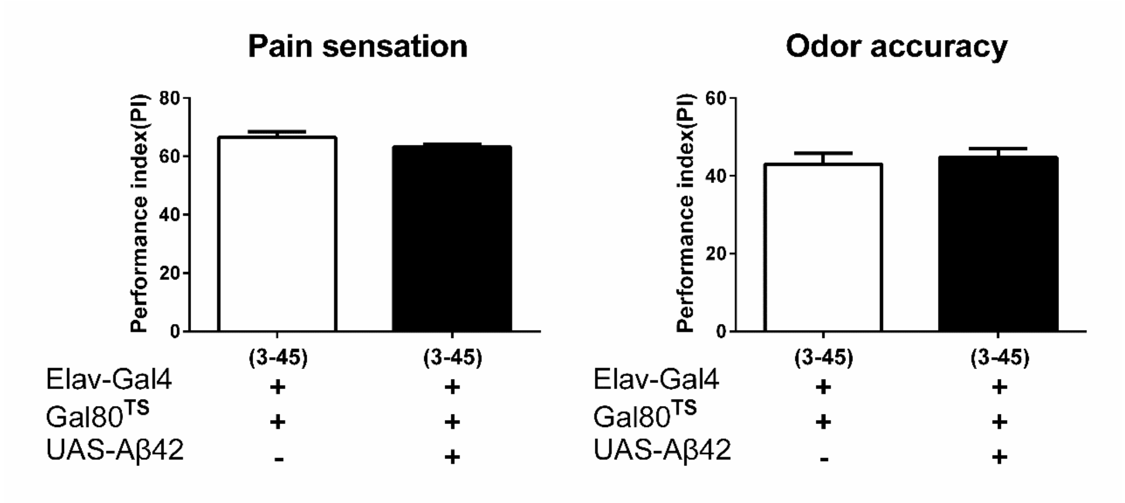

Supplementary Figure 4. Pain sensation and olfactory function are normal in $3_{30^{o}C}$ -$45_{18^{o}C}$ Aβ flies. The normal pain sensation and olfactory function performed in Aβ group of $3_{30^{o}C}$ -$45_{18^{o}C}$ flies, N = 6 respectively.

© 2021. Cheng KC et al. Published online at http://www.aginganddisease.org/EN/10.14336/AD.2021.1015

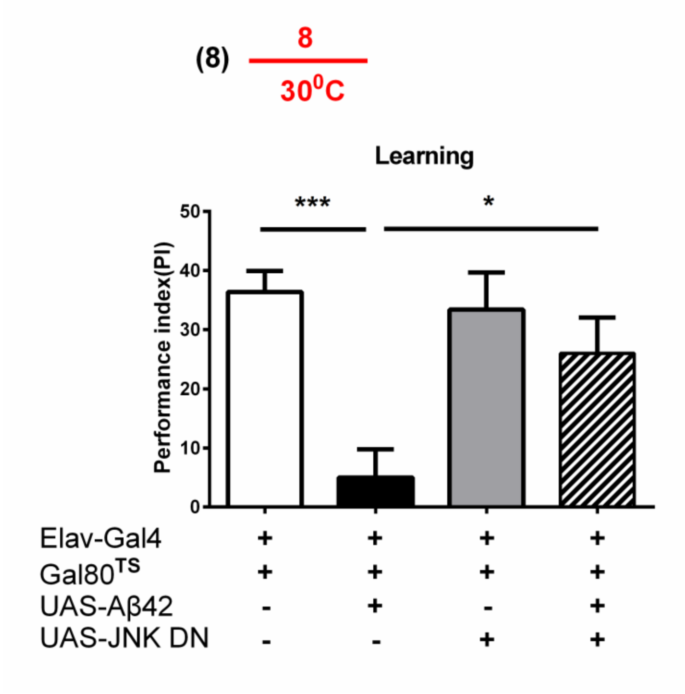

Supplementary Figure 5. Learning impairment was improved by JNK DN overexpression in Aβ flies. Overexpression of JNK DN improved learning impairment in 8dae Aβ flies (Ctrl vs. Aβ42: p = 0.0004, respectively; Aβ42 vs. Aβ42+JNK DN: p = 0.0305, N = 6 respectively). * $p < 0.05$, *** $p < 0.001$. Ctrl represents Elav-Gal4+Gal80^{TS}.

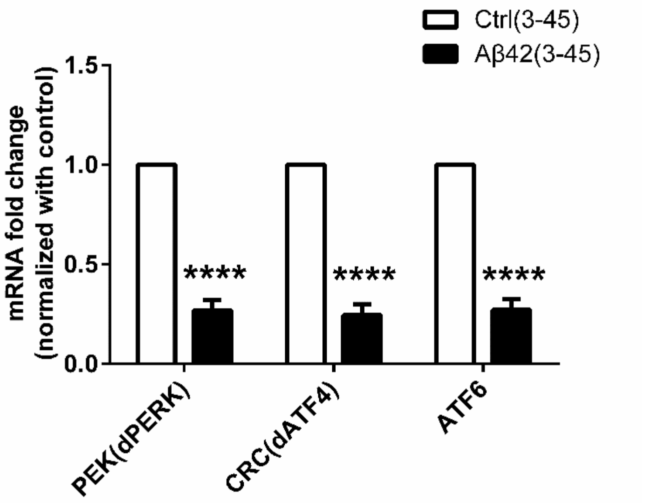

Supplementary Figure 6. The mRNA levels of dPERK, dATF4 and ATF6 were decreased in the 3_{30^oC}-45_{18^oC} Aβ flies. Early Aβ induction for 3days reduced mRNA levels of ER stress effectors in the 3_{30^oC}-45_{18^oC} Aβ flies, N = 6 respectively. **** $p < 0.0001$. All Ctrl represents Elav-Gal4+Gal80^{TS}.

© 2021. Cheng KC et al. Published online at http://www.aginganddisease.org/EN/10.14336/AD.2021.1015

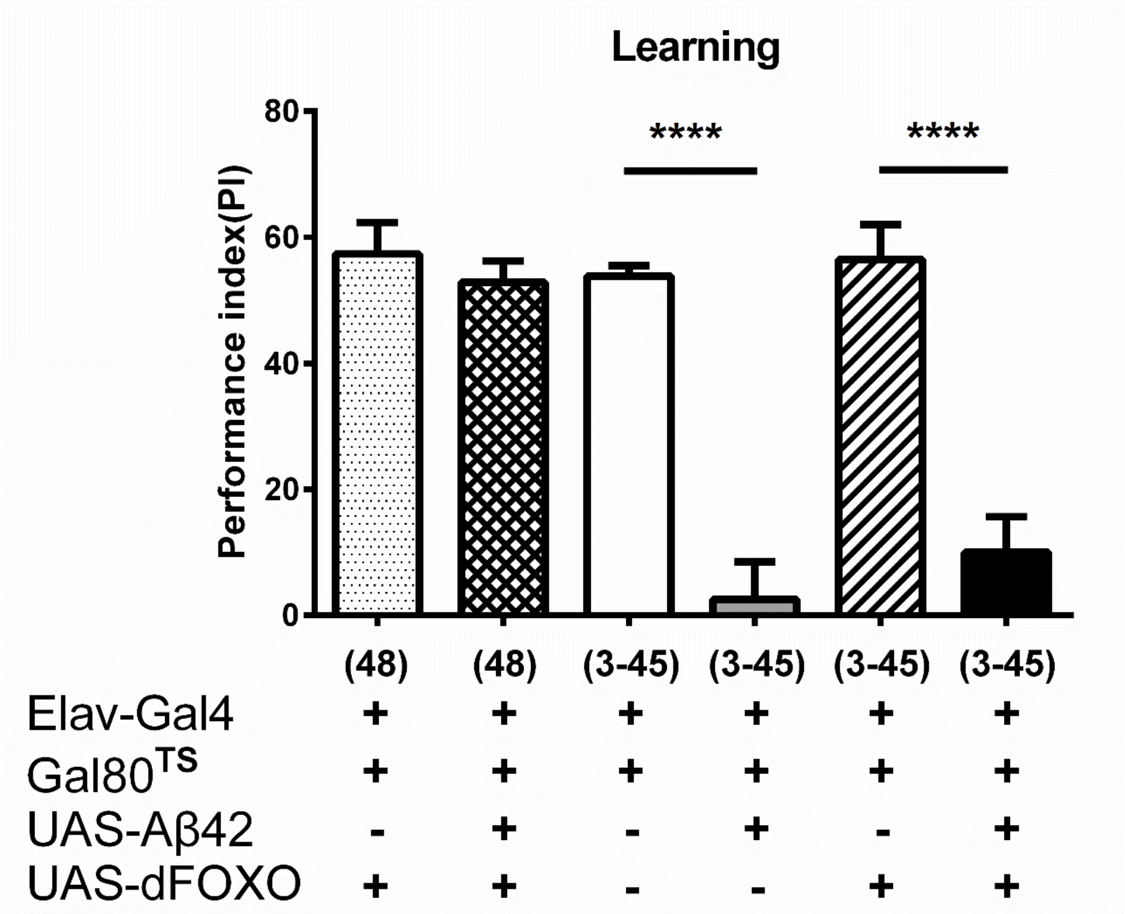

Supplementary Figure 7. FOXO overexpression could not improve learning impairment in $3_{30^{o}C}$-$45_{18^{o}C}$ Aβ flies. Learning impairment was not improved by dFOXO overexpression in Aβ group of $3_{30^{o}C}$ -$45_{18^{o}C}$ flies (Ctrl (3-45) vs. Aβ42 (3-45): $p < 0.0001$ respectively; Aβ42 (3-45) vs. Aβ42+dFOXO (3-45): $p = 0.8752$, N = 6 respectively). **** $p < 0.0001$. ns represents non-significant. Ctrl represents Elav-Gal4+Gal80^{TS}.

© 2021. Cheng KC et al. Published online at http://www.aginganddisease.org/EN/10.14336/AD.2021.1015
